# Differences in management and outcome for colon and rectal carcinoma with synchronous liver metastases: a population‐based cohort study

**DOI:** 10.1111/codi.15468

**Published:** 2020-12-26

**Authors:** Lisen Båverud Olsson, Christian Buchli, Christina Villard, Per J. Nilsson

**Affiliations:** ^1^ Department of Molecular Medicine and Surgery Karolinska Institutet Stockholm Sweden; ^2^ Centre for Digestive Diseases Karolinska University Hospital Stockholm Sweden; ^3^ Department of Medicine Huddinge Unit of Gastroenterology and Rheumatology Karolinska Institutet Stockholm Sweden

**Keywords:** colorectal cancer, synchronous liver metastases, surgery, population‐based

## Abstract

**Aim:**

Surgical treatment of colorectal cancer with synchronous colorectal liver metastases (SCRLM) can follow three different strategies with regard to the timing of liver resection. The aim of this study was to describe the selection of surgical strategy, focusing on differences between colon and rectal cancer with SCRLM, postoperative morbidity/mortality and survival.

**Method:**

This was a retrospective population‐based study of patients with SCRLM registered in the Swedish Colorectal Cancer Registry in the Stockholm/Gotland region during 2010–2017 and treated with surgical resection of the primary tumour and liver metastases (LM). Patients were followed for 5 years or censored at 22 November 2018.

**Results:**

A total of 238 patients met the inclusion criteria during the study period. Patients with rectal cancer were treated with the ‘liver first’ strategy in 70% of cases, whereas the main treatment strategies for colonic tumours were ‘simultaneous resection’ (44%) and ‘primary first’ (37%). Rectal cancer had a superior 5‐year survival rate compared with colon tumours with SCRLM (62 vs. 47%; *p* = 0.033). There was no difference in survival between treatment strategies irrespective of primary tumour location. Postoperative complications occurred most commonly among rectal tumours treated with simultaneous resection (*p* = 0.024).

**Conclusion:**

Patients with rectal cancer and SCRLM were more often treated with the ‘liver first’ strategy than patients with colon cancer. Patients with rectal cancer and SCRLM where both primary tumour and LM were operated on had significantly better survival than corresponding patients with colon cancer.

**What does this paper add to the literature?**

This population‐based study is the first study to date that indicates important differences in both surgical strategy and survival for colon and rectal cancer with synchronous liver metastases. It reveals that rectal cancer with synchronous liver metastases has superior survival with liver first as the ‘standard’ surgical strategy.

## INTRODUCTION

Colorectal cancer is the second most common malignancy worldwide, and around 15% of patients present with synchronous liver metastases (SCRLM) at the time of diagnosis [1, 2]. Surgical resection, often in combination with chemotherapy, can offer long‐term survival, with 5‐year survival in a significant proportion of patients [3]. In recent years, rapid advances have made three surgical strategies available for SCRLM and an increasing proportion of patients can be subject to curative treatment [4]. In addition, radiofrequency ablation (RFA) of liver metastases has become an additional modality for selected patients and is applicable at several timepoints during treatment [5]. However, the optimal timing of surgery is still controversial, with the option of resecting the ‘primary first’ (the classical strategy), the ‘liver first’ or ‘simultaneous resection’.

In the absence of conclusive international or regional guidelines, surgical strategy differs among patients and is decided at multidisciplinary team (MDT) conferences. The liver first strategy has been described to be suitable for rectal cancer and patients with a high liver tumour burden [6, 7]. The preoperative treatment and treatment response differ in important aspects between colon and rectal cancer; rectal cancer patients often receive neoadjuvant (chemo)radiotherapy and have a greater tumour regression in response to therapy [8]. However, in the Swedish Colorectal Cancer Registry (SCRCR) the reported long‐term survival is equivalent for colon and rectal cancer [9, 10].

The present study aimed to describe the selection of surgical strategy for colorectal cancer with SCRLM in the Stockholm/Gotland region where both the primary tumour and liver metastases were resected. The study focuses on differences between colon and rectal cancer, postoperative morbidity/mortality and overall survival.

## METHOD

### Study design and setting

This was a population‐based cohort study that included all patients treated with surgery for colorectal cancer and SCRLM in the Stockholm/Gotland region (2.4 million inhabitants). The incidence of SCRLM in the region during the study period (2010–2017) was 17.3% of the total number of patients with colorectal cancer (1498/8673) (see the SCRCR database [11]). Exposure was the location of the primary tumour (colon versus rectum) and 5‐year overall survival was the primary outcome. Colorectal cancer surgery is performed at five units and liver resections are centralized to Karolinska University Hospital. Patients were identified from the prospectively maintained national SCRCR database with a validated coverage of over 99% of colorectal cancer patients in the Stockholm/Gotland region [9, 10, 12]. The SCRCR is linked to the Swedish Tax Agency to obtain valid data on date of death. The National Quality Registry for Liver and Biliary Cancer (SweLiv), incorporating all patients in Sweden with primary and secondary malignancies of the liver, was used to identify patients who had resection of liver metastases. Ethical approval was obtained from the regional Swedish Ethical Review Authority (EPN 2018/925‐31/2). This study has been reported in accordance with STROBE guidelines.

### Participants

All patients registered in SCRCR/SweLiv and treated with segmental bowel resection and surgery for liver metastases in the Stockholm/Gotland Region from January 2010 to December 2017 were included. Patients with extrahepatic metastases, emergency bowel surgery or RFA of the liver metastases were excluded. The multimodal treatment strategy was discussed at MDT conferences and individualized. The categories of surgical strategy were defined according to the timing of surgery as ‘primary first’, ‘liver first’ or ‘simultaneous resection’. Chemotherapy could be given before the first resection (neoadjuvant) and perioperatively (before and after the liver resection); radiotherapy for rectal cancer was given prior to rectal resection.

### Variables and data sources

Epidemiological and oncological data were retrieved from SCRCR and SweLiv, respectively. Information was completed by medical chart review and postoperative complications with a Clavien–Dindo grade of 3a or above were included. The liver tumour burden score (TBS) was calculated from the number of metastases and the size of largest metastases [TBS^2^ = (maximum tumour diameter in cm)^2^ + (number of lesions)^2^], previously described by Sasaki et al. [13]. The TBS score was categorized using <3, 3 to <9, ≥9 points as cut‐offs [12]. Overall survival was calculated from the date of diagnosis and death from any cause was registered as ‘event’. Follow‐up was censored after 5 years or at 22 November 2018.

### Statistical methods

Register data were analysed using Stata software version 15.0 (Stata Corp LLC). Descriptive data are presented as frequency (proportion) for categorical variables and as median (range) for continuous variables. Groups were compared by Fisher’s exact test or Mann–Whitney *U*‐test as appropriate and a *p*‐value <0.05 was considered statistically significant. Small cell counts and skewed distributions for some covariates were the reasons to compare groups with Fisher’s exact test and Mann–Whitney *U*‐test, respectively. Overall survival is presented with Kaplan–Meier plots and compared with log‐rank tests. Uni‐ and multivariable Cox regression models, stratified for the binary covariate age over 75 years, were applied to analyse the effect of covariates on overall survival. Conservative use of chemotherapy above the age of 75 and death unrelated to colorectal cancer or its treatment were the reasons to stratify the Cox regression models. Apart from location of primary tumour and surgical strategy, covariates that changed point estimates by more than 10% were included in the multivariable model. The proportional hazards assumption was assessed using a log–log plot of survival and Schoenfeld residuals, and no evidence was found to reject the proportional hazard assumption.

## RESULTS

### Participants and baseline characteristics

**FIGURE 1:**
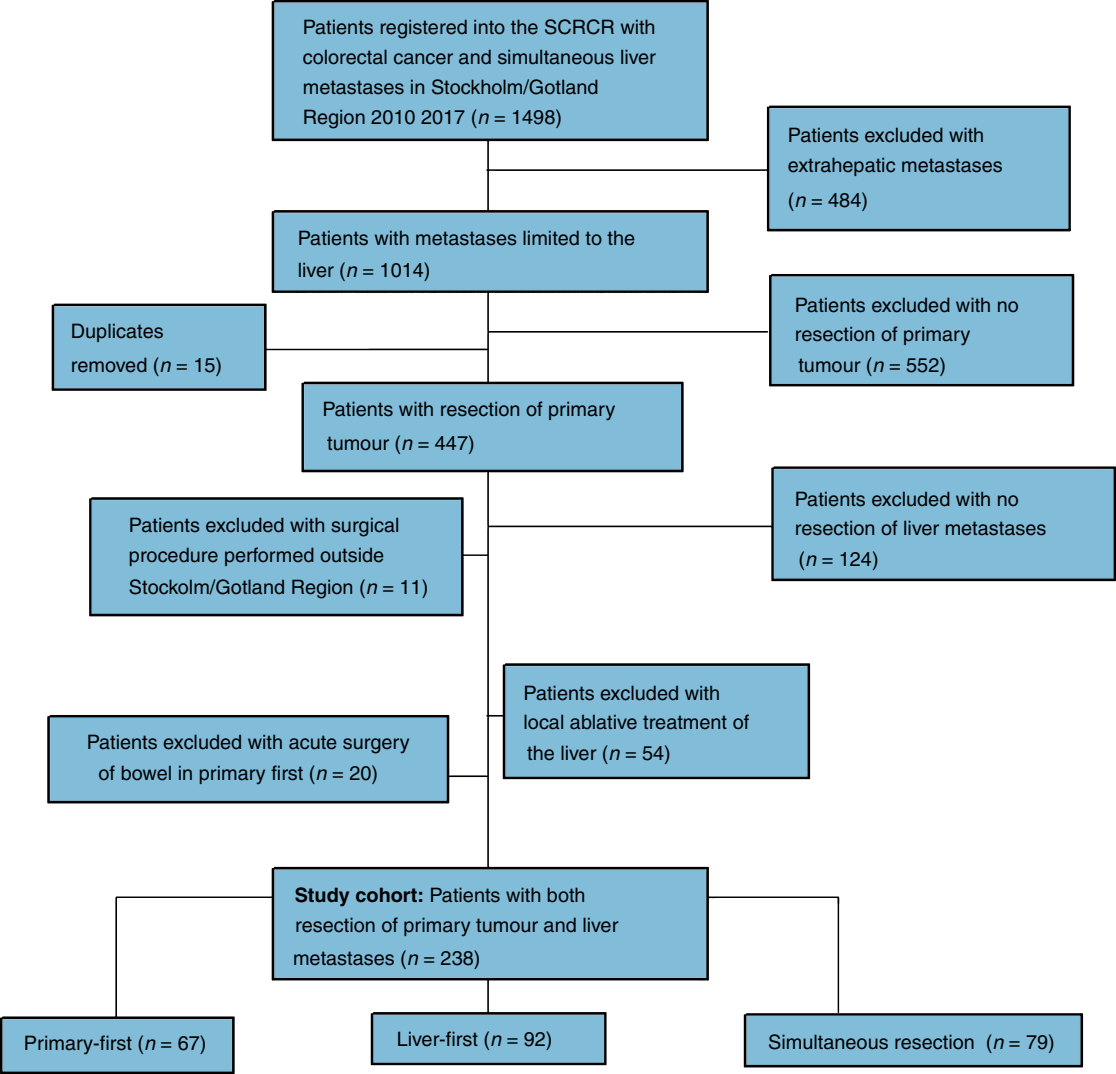
Flow chart of inclusion and surgical strategy in patients with synchronous colorectal liver metastases in Stockholm/Gotland region 2010–2017 (SCRCR, Swedish Colorectal Cancer Registry)

A total of 238 patients had surgery for both primary tumour and liver metastases in the Stockholm/Gotland region between 2010 and 2017. A flow chart of inclusion and surgical strategy is presented in Figure 1. Liver first was the surgical strategy in 39%, with simultaneous resection and primary first being less common, at 33% and 28%, respectively. Descriptive data on patient characteristics and cancer stage are presented in Table 1. The primary tumour location was the colon in 61%. Gender and American Society of Anesthesiologists (ASA) score were comparable between patients with their primary tumour in the colon and the rectum. Clinical TNM classification showed comparable categories for the primary tumour (*p* = 0.521), regional lymph nodes were negative in 33% of patients with colon cancer compared with 15% with rectal cancer (*p* = 0.001). In patients with a primary colon cancer, 75 (52%) of tumours were located on the right side or transverse colon and the remaining 69 (48%) were left‐sided. Tumours in the rectum were distal in 19 (21%) patients, mid in 39 (43%) and proximal in 33 (36%). No significant differences with regard to liver TBS or bilobar engagement were detected between colon and rectal cancer. The extent of liver disease was comparable as assessed by TBS (median 3.6 vs. 3.4, *p* = 0.955) and the proportion of bilobar engagement (39% vs. 45%, *p* = 0.413).

**TABLE 1:** Characteristics of 238 patients with colorectal cancer and synchronous liver metastasis treated with surgical resection of both primary tumour and liver metastases

Characteristics of patients	No. (%) of patients			
	Colon	Rectum		*p* ^d^
No. of patients	146 (61)^c^	92 (39)		
Age (years)^a^	67 (35–88)	64 (36‐86)		0.035^e^
Gender				0.096
Female	57 (39)	26 (28)		
Male	89 (61)	66 (72)		
ASA classification				0.812
I	18 (12)	15 (16)		
II	72 (49)	46 (50)		
III	52 (36)	29 (32)		
IV	4 (3)	2 (2)		
Clinical category primary tumour				0.521
T1–2	12 (8)	9 (10)		
T3	86 (60)	54 (59)		
T4	30 (21)	27 (29)		
Tx^b^	16 (11)	2 (2)		
Clinical category lymph nodes				0.001
N0	49 (33)	14 (15)		
N1–2	89 (61)	77 (84)		
Nx^b^	9 (6)	1 (1)		
Pathological category primary tumour				<0.001
T0	1 (1)	9 (10)		
T1–2	20 (14)	16 (17)		
T3	70 (48)	56 (61)		
T4	54 (37)	10 (11)		
Tx^b^	0	1 (1)		
Pathological category lymph nodes				0.001
N0	46 (32)	43 (47)		
N1	58 (40)	38 (41)		
N2	41 (28)	9 (10)		
Nx^b^	0	2 (2)		
Bilobar liver disease	54 (39)	41 (45)		0.413
Liver TBS^a^	3.6 (1.0‐19)	3.4 (1.1‐17)		0.955^e^

Abbreviations: ASA, American Society of Anesthesiologists; TBS, tumour burden score.

^a^ Continuous variables as median (range).

^b^ x means incomplete data or impossible to pretherapeutically stage.

^c^ In one patient, after completing both surgical procedures, the primary tumour and metastases were assessed to be originating from a gastric carcinoma.

^d^ Fisher’s exact test was used for all categorical variables.

^e^ Mann–Whitney *U*‐test for continuous variables.

### Surgical strategy

Data on surgical strategy and the use of neoadjuvant chemo‐ and/or radiotherapy for colon and rectal primary tumours with SCRLM are presented in Table 2. The selection of surgical strategy differed significantly between primary colon and rectal tumours. A liver first strategy was applied in 70% of rectal cancer patients whereas patients with a primary colon tumour most commonly underwent a simultaneous resection (44%) or primary first strategy (37%) (*p* < 0.001) (Table 2). Differences in liver TBS between the surgical strategies within primary colon and rectal tumours were observed. For colon primary tumours, the median liver TBS was 4.2 vs. 6.1 vs. 2.4 for primary first, liver first and simultaneous resection, respectively (*p < *0.001). For rectal primary tumours the median liver TBS was 2.6 vs. 4.2 vs. 2.0 for primary first, liver first and simultaneous resection, respectively (*p < *0.001).

**TABLE 2:** Surgical strategy and radio‐/chemotherapy for 238 patients with colorectal cancer and synchronous liver metastasis treated with surgical resection of both primary tumour and liver metastases

(a)	Colon, *n* (%)	Rectum, *n* (%)	*p* ^b^
No. of patients	146	92	
Surgical strategy			<0.001
Primary first	54 (37)	13 (14)	
Liver first	28 (19)	64 (70)	
Simultaneous resection	64 (44)	15 (16)	
Radiotherapy +^a^	–	84 (91)	‐
Neoadjuvant chemotherapy	71 (50)	69 (76)	<0.001
Perioperative chemotherapy	100 (70)	72 (79)	0.131
No. of cycles^b^	12 (3‐12)	12 (5‐15)	0.103^c^

(b)	Colon, *n* (%)				Rectum, *n* (%)			*p*
Based on surgical strategy	PF	LF	SR	*p*	PF	LF	SR	
Neoadjuvant chemotherapy	12 (22)	26 (93)	33 (54)	<0.001	4 (33)	55 (86)	10 (67)	<0.001
Perioperative chemotherapy	41 (76)	26 (93)	33 (54)	<0.001	7 (58)	55 (86)	10 (67)	0.038

Abbreviations: LF, liver first; PF, primary first; SR, simultaneous resection.

^a^ In patients with rectal cancer.

^b^ Continuous variables as median (range).

^c^ Mann–Whitney *U*‐test for continuous variables.

Data on the use of chemotherapy for primary colon and rectal tumours, respectively, are shown in Table 2. Patients received 5‐fluorouracil (5‐FU)‐based chemotherapy, in general combined with oxaliplatin. Only a few (15%) received bevacizumab, an antivascular endothelial growth factor therapy in combination with 5‐FU‐based chemotherapy. The median total number of cycles was 12 for both groups (*p *= 0.103). Patients with rectal cancer received neoadjuvant chemotherapy more often than colon cancer patients (76% vs. 50%, respectively; *p* < 0.001). Neoadjuvant chemotherapy was given more often to liver first than to primary first and simultaneous resection patients.

### Short‐term postoperative outcomes

Length of stay (LoS) and other short‐term postoperative outcomes are presented in Table 3.

**TABLE 3:** Short‐term postoperative outcomes in 238 patients with colorectal cancer and synchronous liver metastasis after resection of primary tumour and liver metastasis

	Colon, *n* (%)	Rectum, *n* (%)	*p* ^d^
No. of patients	146	92	
LoS (days)^a^	13 (3‐85)	16.5 (2–128)	<0.001^e^
C–D grade >3a^b^	45 (31)	30 (33)	0.886
Reoperation	23 (16)	11 (12)	0.453
Care in the ICU	13 (9)	4 (4)	0.208
LoS in ICU (days)^a^	3 (1–8)	4.5 (1–61)	0.697^e^
Readmission^c^	25 (17)	24 (26)	0.102
90‐day mortality	3 (2)	0 (0)	0.286
Preresection stoma	35 (24)	24 (26)	0.758
Radical resection	108 (89)	74 (90)	0.819

Abbreviations: C–D, Clavien–Dindo; ICU, intensive care unit; LoS, length of stay.

^a^ Continuous variables as median (range).

^b^ Number (%) of patients that had one or more complications with Clavien–Dindo grade 3a or above after bowel or liver surgery.

^c^ Readmission to hospital within 30 days of discharge.

^d^ Fisher’s exact test was used for all categorical variables.

^e^ Mann–Whitney *U*‐test for continuous variables.

Median LoS including both surgical procedures (when staged) for colon cancer patients was 13 days compared with 16.5 days for rectal cancer (*p* < 0.001). Postoperative morbidity, mortality, ICU stay and readmission rate did not differ between both groups, with an overall complication rate (Clavien–Dindo grade 3a or above) of 32% and 90‐day mortality of 1.3%. The complication rate stratified on surgical strategy was higher for simultaneous resection (53%) than primary first (8%) and liver first (33%) in patients with rectal cancer (*p* = 0.033), but similar for the three surgical strategies in patients with colon cancer (*p* = 0.500) (Table S1 in the online Supporting Information). A defunctioning stoma was more frequently used in colon cancer patients (60%) treated with the liver first strategy compared with other strategies (*p* < 0.001) (Table S1).

### Overall survival

**FIGURE 2:**
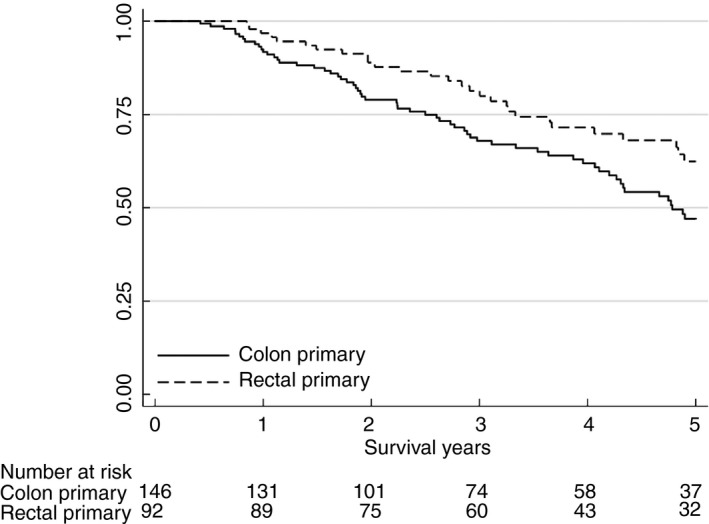
Overall survival for 238 patients with synchronous colorectal liver metastasis after resection of primary tumour and liver metastasis, *p* = 0.033 (log‐rank test)

The median follow‐up time was 42 months. The median overall survival for the entire group of patients was 62.4 months (95% CI 57.3–74.1) with a 5‐year survival rate of 53%. The number of deaths during follow‐up was 89, 61 for colon cancer patients and 28 for rectal cancer, with a crude incidence ratio of 0.13 vs. 0.08, respectively (incidence rate ratio 0.64, 95% CI 0.39–1.02, *p *= 0.047). There was no difference in survival between treatment strategies irrespective of primary tumour location. The proportion of recurrent metastatic disease during follow‐up was not statistically significantly different between colon and rectal cancer patients (65% vs. 58%, *p = *0.403). The 5‐year overall survival was significantly lower for colon cancer (47%) compared with rectal cancer (62%) (*p *= 0.033) (Figure 2). The unadjusted hazard rate ratio (HR) for overall survival was 0.64 (0.41–1.00, *p* = 0.052) for patients with rectal cancer compared with colon cancer, and liver TBS was a significant predictor (Table 4). The Cox model adjusted for bilobar liver disease and surgical strategy confirmed this difference, with a HR of 0.54 (95% CI 0.32–0.92, *p *= 0.022). Categorization of location of primary tumour in right/transverse colon, left colon and rectum showed a gradually increased survival for primary tumours of the distal part of the large bowel with HR 0.64 (95% CI 0.38–1.08, *p *= 0.094) for the left colon and HR 0.49 (95% CI 0.30–0.82, *p *= 0.006) for the rectum compared with right‐sided cancers.

**TABLE 4:** Uni‐ and multivariate Cox proportional hazards model analysis of overall survival for 238 patients with colorectal cancer and synchronous liver metastasis after resection. The analysis was stratified for the binary covariate age over 75 years

	Univariate HR (95% CI)	*p*	Multivariate HR (95% CI)	*p*
Primary tumour				
Colon	Ref.		Ref.	
Rectum	0.64 (0.41–1.00)	0.052	0.54 (0.32–0.92)	0.022
Primary tumour localization				
Right and transverse colon	Ref.			
Left colon	0.64 (0.38–1.08)	0.094		
Rectum	0.49 (0.30–0.82)	0.006		
Gender				
Male	Ref.			
Female	1.32 (0.86–2.01)	0.203		
ASA score				
1	Ref.			
2	1.05 (0.55–2.00)	0.892		
3–4	1.52 (0.77–3.00)	0.231		
Bilobar liver disease				
No	Ref.		Ref.	
Yes	1.71 (1.11–2.63)	0.016	1.73 (1.09–2.75)	0.019
Liver TBS				
<3	Ref.			
>3 to <9	1.88 (1.13–3.13)	0.014		
>9	3.62 (1.88–7.00)	<0.001		
Surgical strategy				
Primary first	Ref.		Ref.	
Liver first	1.06 (0.63–1.78)	0.831	1.35 (0.74–2.44)	0.328
Simultaneous resection	1.10 (0.65–1.84)	0.724	1.13 (0.66–1.92)	0.662

Abbreviations: ASA, American Society for Anesthesiologists; HR, hazard rate ratio; TBS, tumour burden score.

## DISCUSSION

This population‐based study shows that for patients with rectal cancer and SCRLM a liver first strategy was applied in more than two thirds of patients whereas no clear pattern for strategy selection could be detected among patients with colon cancer and SCRLM. Furthermore, patients with rectal cancer were shown to have a significantly improved overall survival compared with the corresponding colon cancer patients with SCRLM, also in a multivariable Cox analysis. Clinical pathways differ between colon and rectal cancer with SCRLM, including preoperative imaging and neoadjuvant treatment, rendering direct comparison of clinical and pathological staging impossible. However, the similar liver TBS between colon and rectal tumours is an indication of a comparable stage for patients in the two groups. The superior results for rectal cancer patients with SCRLM reported herein may be at least partly dependent on these patients being younger and receiving more neoadjuvant therapy, but clear differences in the selection process appear to exist.

The surgical strategy chosen for rectal cancer patients with SCRLM was mainly a liver first strategy (70%), which has been suggested to be favourable by a consensus recommendation [7]. The liver first strategy has the benefit of giving rectal cancer patients longer to respond to treatment of the primary tumour and, in the presence of extensive liver disease, starting systemic and surgical treatment without delay for liver metastases [14]. This was reflected in the present study, where patients irrespective of primary tumour location, operated on with a liver first strategy had the highest median liver TBS and received most neoadjuvant chemotherapy (88%). Liver TBS was shown in the univariate Cox analysis to be a significant predictor of survival. However, there was no difference in median liver TBS between patients with colon and rectal cancer. In Sweden most patients with rectal cancer and SCRLM are also treated with neoadjuvant radiotherapy delivered as a short course (5 × 5 Gy) after chemotherapy and immediately prior to liver surgery. Unlike for rectal cancer, recommendations for one strategy in colon cancer patients with SCRLM are absent [6]. Furthermore, it is difficult to see a clear pattern regarding strategy selection, even when taking ASA score, age, stage of primary tumour and extent of metastatic burden into account. Use of the liver first strategy is still limited for colon cancer patients with SCRLM in the present study and most patients were treated with simultaneous resection (44%) or the primary first strategy (37%).

Simultaneous resection has the benefit of a considerably shorter LoS for both colon and rectal primary tumours, as reported by other studies [15, 16]. However, a higher risk of major complications for simultaneous resection has been debated, especially for rectal primary tumours and when the hepatic resection is major [17, 18]. Irrespective of the surgical strategy selected, no major differences in postoperative complications were found when analysed for all patients. However, it is noteworthy that the highest rates of Clavien–Dindo complications ≥3a, readmission and reoperation were found in the small number of patients (*n* = 15) with rectal cancer who underwent simultaneous resection. Comparison of complication rates between strategies may be hampered by the fact that around one third of patients planned for staged resection progress or have complications after the first surgical procedure that prevent the second step of intended surgical treatment [19, 20]. Despite these observations regarding complication rates, it appears that simultaneous resection with only one in‐patient episode reduces the LoS. Furthermore, for a fair comparison of complications it should be noted that a pretherapeutic defunctioning stoma, more commonly used in colon cancer patients treated with the liver first strategy, carries a risk of affecting quality of life as well as a risk of complications when it is reversed.

The 5‐year overall survival for rectal cancer with SCRLM of 62% is encouraging and comparable to single‐centre studies [21, 22]. Previous studies have reported an inferior outcome in right‐sided colorectal tumours compared with left‐sided tumours including rectal tumours [4, 23, 24], and this has been attributed to a more aggressive tumour biology and later diagnosis [23, 25]. In this study, we chose to report on colon and rectal cancer separately because of the different clinical pathways. However, in the univariate Cox analysis the same relationship of worse survival for right‐sided colorectal cancer was observed. The differences in survival reported in this study may be due to several factors, including a clear treatment strategy for rectal cancers, the choice of surgical strategy, radiotherapy‐induced tumour downstaging in rectal cancer, neoadjuvant chemotherapy or a more favourable tumour biology in left‐sided colorectal tumours. Meta‐analysis of previous studies has not reported superior survival in any surgical strategy [26], and neither could this be seen in the present study. However, in one randomized controlled trial superior survival was seen for simultaneous resection when analysed for all patients starting treatment, partly explained by disease progression before liver resection in one third of patients assigned to a primary first strategy [27].

There are several limitations to this study. Firstly, it is a retrospective observational study and patients were actively selected to one of the surgical strategies, possibly based on factors not revealed by the data collected. Secondly, a positive selection bias may exist, in particular among rectal cancer patients, since tumour progression during neoadjuvant therapy may have led to a decision not to operate. Thirdly, detailed data on perioperative chemotherapy regimen and compliance are lacking, although no bias with respect to colon or rectal cancer should exist. A strength of this study is that it reports strategy selection and outcome, based on validated registers, in a population‐based setting with centralized liver surgery.

## CONCLUSION

In conclusion, the selection of surgical strategy differs between patients with primary rectal cancer and colon cancer and SCRLM. Patients with rectal cancer in whom both primary tumour and liver metastases were operated on were also observed to have significantly better overall survival than patients with colon cancer and SCRLM. Although several factors may have contributed, the lack of a clear strategy for colon cancer patients could have been of importance. While randomized controlled trials may be difficult to perform, prospective algorithm‐based observational studies with a focus on management strategies and possibly increased rates of the liver first strategy for colon cancer patients with SCRLM may be a way forward.

## CONFLICT OF INTEREST

LBO, CB, CV and PJN declare that they have no declarations of interest.

## ETHICAL APPROVAL

Ethical approval was obtained from the regional Swedish Ethical Review Authority (EPN 2018/925‐31/2).

## AUTHOR CONTRIBUTIONS

PJN conceived of the presented idea and supervised the project. LBO and CV collected the data, LBO conducted the data analysis and all authors interpreted the data. LBO and CB were responsible for the statistical analysis. LBO, CB, CV and PJN drafted and revised the manuscript. All authors reviewed the manuscript and approved the final version.

## Funding information

No funding to declare.

## Data Availability

The data that support the findings of this study are available from the corresponding author upon reasonable request.
