# Supplementary material for: Differences in management and outcome for colon and rectal carcinoma with synchronous liver metastases: a population‐based cohort study
Source: Colorectal Dis. 2020 Dec 26;23(4):860–7. doi: 10.1111/codi.15468 (PMC8246906; doi:10.1111/codi.15468)
Supplement: Supplementary file 1 [file CODI-23-860-s001.docx]

**Supplementary Table 1. Short-term postoperative outcomes** stratified for primary tumour and surgical strategy in 238 patients with colorectal cancer and synchronous liver metastasis after resection of primary tumour and liver metastasis.

|  | **Colon** | | | | **Rectum** | | | |
| --- | --- | --- | --- | --- | --- | --- | --- | --- |
|  | PF | LF | SR | *P †* | PF | LF | SR | *P †* |
| No. of patients | 54 | 28 | 64 |  | 13 | 64 | 15 |  |
| LoS, days^a^ | 15  (4–40) | 14  (5–85) | 8  (3–37) | <0.001 | 17  (9–27) | 18  (9–128) | 10  (2–22) | <0.001 |
| C-D grade >3a^b^ | 15 (28) | 11 (41) | 19 (30) | 0.500 | 1 (8) | 21 (33) | 8 (53) | 0.033 |
| Re-operation | 8 (15) | 4 (14) | 11 (17) | 0.956 | 0 (0) | 7 (11) | 4 (27) | 0.110 |
| Care in the ICU | 4 (7) | 4 (14) | 5 (8) | 0.520 | 0 (0) | 3 (5) | 1 (7) | 0.773 |
| LoS in ICU, days^a^ | 2  (1–6) | 6  (4–8) | 3  (1–6) | 0.333 | - | 7  (1–61) | 2 | 0.655 |
| Readmission^c^ | 10 (19) | 5 (18) | 10 (16) | 0.881 | 2 (15) | 15 (23) | 7 (47) | 0.121 |
| 90-day mortality | 1 (2) | 0 (0) | 2 (3) | 1.000 | 0 (0) | 0 (0) | 0 (0) | 1.000 |
| Pre-resection stoma | 8 (15) | 16 (57) | 11 (17) | <0.001 | 1 (8) | 19 (30) | 4 (27) | 0.267 |
| Radical resection | 41 (89) | 20 (91) | 47 (87) | 1.000 | 10 (83) | 52 (91) | 12 (92) | 0.731 |

^a^Continuous variables as median (range). ^b^Number (%) of patients that had ≥1 complication with Clavien-Dindo grade 3a or above after bowel or liver surgery. ^c^Readmission to hospital within 30 days of discharge. *†*Fisher’s exact test was used for all categorical variables. §Kruskal-Wallis test or Mann-Whitney U-test for continuous variables. Abbreviations: PF, Primary first; LF, Liver first; SR, Simultaneous resection; LoS, Length of stay; C-D, Clavien-Dindo; ICU, Intensive care unit.
